# Tropical Plant Extracts Modulating the Growth of *Mycobacterium ulcerans*

**DOI:** 10.1371/journal.pone.0124626

**Published:** 2015-04-23

**Authors:** Benjamin Mougin, Roger B. D. Tian, Michel Drancourt

**Affiliations:** URMITE (Unité de Recherche sur les Maladies Infectieuses et Tropicales Emergentes), UMR CNRS 7278, IRD 198, Inserm 1095, Aix Marseille Université, Marseille, France; The University of Hong Kong, HONG KONG

## Abstract

*Mycobacterium ulcerans*, the etiologic agent of Buruli ulcer, has been detected on aquatic plants in endemic tropical regions. Here, we tested the effect of several tropical plant extracts on the growth of *M*. *ulcerans* and the closely related *Mycobacterium marinum*. *M*. *ulcerans* and *M*. *marinum* were inoculated on Middlebrook 7H11 medium with and without extracts from tropical aquatic plants, including *Ammannia gracilis*, *Crinum calamistratum*, *Echinodorus africanus*, *Vallisneria nana* and *Vallisneria torta*. Delay of detection of the first colony and the number of colonies at day 7 (*M*. *marinum*) or day 16 (*M*. *ulcerans*) were used as endpoints. The first *M*. *ulcerans* colonies were detected at 8 ± 0 days on control Middlebrook 7H11 medium, 6.34 ± 0.75 days on *A*. *gracilis*-enriched medium (p<0.01), 6 ± 1 days on *E*. *africanus*- and *V*. *torta*-enriched media (p<0.01), 6 ± 0 days on *V*. *nana*-enriched medium (p<0.01) and 5.67 ± 0.47 days on *C*. *calamistratum*-enriched medium (p<0.01). Furthermore, the number of detected colonies was significantly increased in *C*. *calamistratum*- and *E*. *africanus*-enriched media at each time point compared to Middlebrook 7H11 (p<0.05). *V*. *nana*- and *V*. *torta*-enriched media significantly increased the number of detected colonies starting from day 6 and day 10, respectively (p<0.001). At the opposite, *A*. *gracilis*-enriched medium significantly decreased the number of detected colonies starting from day 8 PI (p<0.05). In conclusion, some aquatic plant extracts, could be added as adjuvants to the Middlebrook 7H11 medium for the culturing of *M*. *marinum* and *M*. *ulcerans*.

## Introduction

Buruli Ulcer is the third most common mycobacteriosis in the world [1]. It is responsible for skin ulcers that lead to subcutaneous and bone infections and eventually causes necrosis with potentially debilitating scars and amputations [1]. This difficult-to-treat infection is endemic in tropical regions of 33 countries, mainly within Central and Western Africa, in Victoria and Queensland states in Australia and at lower rates in South America and Asia [1]. Buruli ulcer is caused by the acid-fast bacilli *Mycobacterium ulcerans*; genomic analysis of *M*. *ulcerans* indicated that it emerged from *Mycobacterium marinum* [2,3] after the acquisition of a mycolactone-toxin-encoding plasmid [4]. *M*. *marinum* is an aquatic mycobacterium hosted by ectothermic animals, mainly fish [5,6]. After its inoculation, *M*. *marinum* causes skin granulomas, including vivarium granuloma [7,8].

The laboratory diagnosis of Buruli ulcer relies on the PCR-based detection of *M*. *ulcerans*-specific sequences, including IS*2404*, IS*2606* and ketoreductase-B domain of the mycolactone polyketide synthase genes [9]. Isolation and culture of *M*. *ulcerans* is not a routine technique for the diagnosis of Buruli ulcer, as *M*. *ulcerans* is a slow-growing organism with a doubling time of more than 30 hours. The reservoir, sources and routes of transmission of *M*. *ulcerans* remain elusive, although epidemiological observations suggest stagnant aquatic environments as potential reservoirs [10,11] and insect bites as an additional risk factor [11]. Interestingly, some free-living aquatic plants stimulate the growth of *M*. *ulcerans* [12]. Also, the presence of *M*. *ulcerans* DNA was significantly associated with specific communities of submerged terrestrial plants, in Ghana [13]. Recently, *M*. *ulcerans* DNA was detected in stagnant water covering decaying organic materials [14]. However, only one fully characterized *M*. *ulcerans* isolate has been isolated from an environmental source, and none have been isolated from plants [15]. Later environmental isolate has been made from an aquatic Hemiptera, genus *Gerris* collected in Benin, after inoculation of liquid Middlebrook medium, three passages in mouse footpad and subculture on Löwenstein-Jensen medium [15].

In this study, we further explored whether the growth of *M*. *ulcerans* and *M*. *marinum* was modulated by aquatic plants prevalent in some Buruli ulcer regions. We grew mycobacteria in Middlebrook 7H11-based medium complemented with plant extracts and monitored the growth of mycobacterial colonies using autofluorescence of bacilli.

## Methods

### Mycobacterial strains

An *M*. *marinum* clinical isolate [8] was identified by *rpoB* gene sequence analysis [16]. *M*. *ulcerans* ATCC19423^T^ strain Agy99 (classical lineage) [17] was isolated in 1999 in Ghana (Ga District, Great Accra Region) from the biopsy of an ulcerative lesion on the right elbow of a female patient. Identification was confirmed by using a multiplex real-time PCR as previously described [9]. To produce a large enough inoculum, both isolates were cultured at 32°C in Middlebrook 7H9 liquid medium (Becton Dickinson, Le Pont de Claix, France) complemented with oleic acid, bovine albumin, dextrose, and catalase enrichment (OADC, Becton Dickinson). The *M*. *ulcerans* isolate was manipulated in a Biosafety Level 3 laboratory, Medical School, Marseilles, France. After production in broth, cultured mycobacteria were passed three times through a 30-gauge needle in order to disperse aggregates, and mycobacteria were suspended in phosphate buffered saline (PBS, pH 6.5) in a tube containing glass beads. The tube was vigorously vortexed in order to further separate any bacterial aggregates. The suspension was calibrated at 10^5^ mycobacteria/mL by observing and counting dispersed bacteria after Ziehl-Neelsen staining and by measuring the optical density of the suspension (Cell Density Meter Model 40 spectrophotometer, Fischer Scientific, Illkirch, France). Inoculum was expressed in mycobacteria / mL.

### Comparing naked eye and autofluorescence detection of colonies

For each *M*. *marinum* and *M*. *ulcerans* strain, 100 μL of a 10^5^ mycobacteria/mL bacterial suspension were seeded on a plate containing Middlebrook 7H11 medium, incubated at 32° C. Negative control plates were inoculated with sterile PBS. Two days, four days, eight days and sixteen days after inoculation, colonies were observed both by the naked-eye and using a MZ-FLIII fluorescence microscope (Leica, Nanterre, France) equipped with a GFP filter and an ICA digital camera (Leica) to detect mycobacterial autofluorescence. Counting of fluorescent colonies was performed using the Leica Application Suite software (Leica). Experiments were performed in three replicates.

### Plant extract-enriched culture media

A total of 19 g of Middlebrook 7H11 agar powder (Becton Dickinson) was mixed with 10 mL of 50% glycerol (Sigma-Aldrich, Saint-Quentin Fallavier, France) and suspended in 885 mL of water. The mixture was autoclaved at 121°C for 10 min, and 5 mL of a 0.22 μm-filtered 200 g/L tryptone solution (Becton Dickinson) and 100 mL OADC supplement were added to obtain 1 L of base medium. Tropical aquatic plants (Paonefish, Bezons, France) were selected based on the widespread growth in Buruli ulcer-endemic regions and for requiring growth conditions compatible with those of *M*. *ulcerans* (Table 1) (S1 File). Then, 50 g of fresh plants was washed in sterile distilled water for 5 min, crushed in a sterile mortar, suspended in a final volume of 500 mL of water and incubated at 4°C overnight. The resulting solution was filtered through a 330 μm porosity grid, then autoclaved at 121°C for 10 minutes. The preparation was successively filtered through a 20 μm-pore membrane, then a 0.22 μm-pore membrane. This filtrate was added at 10% final concentration (vol:vol) to Middlebrook 7H11 base medium maintained at 50°C, and the final medium was poured into sterilized 55-mm diameter Petri dishes (Becton Dickinson). Middlebrook 7H11 medium completed with 10% (vol:vol) filtered PBS was used as control. A 100μL-volume of a 2.5 x 10^5^ mycobacterial/mL in PBS was inoculated in parallel onto a control Middlebrook 7H11 plate and onto a plant extract-enriched plate. Plates were then incubated at 32°C in an Anaerogen jar (Oxoid, Dardilly, France) in presence of Campygen microaerophilic atmosphere generators (Oxoid). Negative control plates inoculated with sterile PBS were incubated in parallel in the ratio of one negative control plate for six inoculated plates. Cultures were observed by autofluorescence and naked-eye every day for seven days for *M*. *marinum* and every two days for 16 days for *M*. *ulcerans* in order to numerate the colonies. The counting of fluorescent colonies was performed using Leica Application Suite software (Leica). Colonies were identified using matrix-assisted laser desorption/ionization mass spectrometry (MALDI-TOF-MS) [18]. All experiments were performed in six replicates. Delay of detection of the first colony and the number of colonies at day seven (*M*. *marinum*) or day 16 post-inoculation (*M*. *ulcerans*) were used as two endpoints to compare the growth of mycobacteria on Middlebrook 7H11 medium with and without tropical aquatic plant extracts.

**Table 1:** Tropical aquatic plants used in this study to enrich Middlebrook 7H11 solid culture medium.

Common name	Scientific name	Environment	Distribution	Growth temperature
Ammannia	*Ammannia gracilis*	Sandy edges of rivers, swamps	Western Africa	22–30° C
Cameroon Crinum	*Crinum calamistratum*	Freshwater	Central Africa	20–28° C
African Amazon sword	*Echinodorus africanus*	Freshwater	Africa (Cameroon), South Africa	20–32° C
Vallisneria nana	*Vallisneria nana*	Shallow edges of rivers	Australia (State of Queensland)	20–28° C
Corkscrew Vallis	*Vallisneria torta*	Freshwater	Japan, South-East Asia	19–30° C

### Statistics

The Two-Sample Independent t-Test was used to compare delays of positivity and kinetics of growth. A *p* value of <0.05 was considered significant.

## Results

### Autofluorescence validation

Negative control plates remained free of colonies by naked-eye and autofluorescence reading. As for *M*. *marinum*, naked-eye and autofluorescence detected no colony at day 2, 10.6±1.5 versus 30±2 colonies at day 4 (P<0.05), and 67.3±2 versus 133±2.64 colonies at day 8 (P<0.05). Colonies were too numerous to be counted at day 16. As for *M*. *ulcerans* ATCC 19423^T^, the same comparison yielded no colonies at day 2 and day 4, 0 versus 35±3.6 colonies at day 8 (P<0.05) and 124±4.16 versus 247.67±5.68 colonies at day 16.

**Fig 1:**
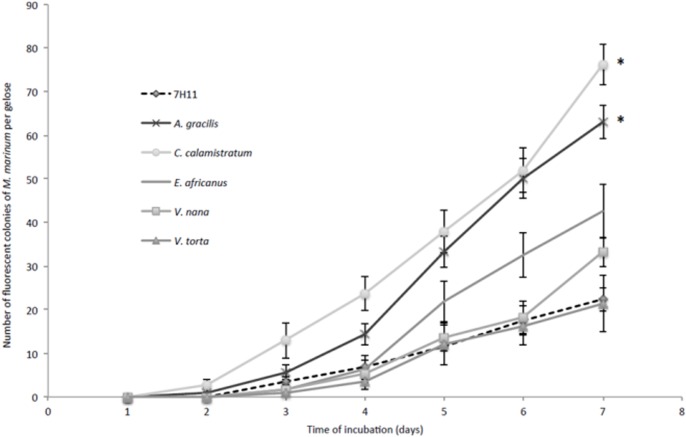
*A*. *gracilis* and *C*. *calamistratum* extracts increase the growth rate of *M*. *marinum* on Middlebrook 7H11-enriched media. Kinetics of growth of *M*. *marinum* in Middlebrook 7H11 medium and Middlebrook 7H11-based solid medium enriched with plant filtered extracts (10%, vol:vol), detected by autofluorescence. Each data point represents the mean ± standard error for six plates per time point. The asterisks represent medium facilitating a significant increase of the number of *M*. *marinum* colonies at each time point compared to Middlebrook 7H11 medium.

Further, all plant extract-enriched, non-inoculed media presented an autoflorescence whose intensity varied depending on the vegetal. All colonies of *M*. *marinum* and *M*. *ulcerans* were observed as spontaneously fluorescent, independently from the medium. The fluorescence system could detect 30 μm-diameter colonies which were undetectable by naked eye. Autofluorescence significantly reduced (p<0.001) the delay of mycobacteria colony detection in Middlebrook 7H11 medium and in all tested vegetal-enriched media (Fig 1).

### Delay of detection of the first colony

Using autofluorescence, the delay of detection of the first *M*. *marinum* colonies was 2.84 ± 0.39 days on Middlebrook 7H11, 2.51 ± 0.46 days on *A*. *gracilis*-enriched medium (p<0.05) and 2.19 ± 0.37 days on *C*. *calamistratum*-enriched medium (p<0.03). All plant extracts significantly decreased the delay of *M*. *ulcerans* colony detection compared to Middlebrook 7H11 medium alone. The delay of detection of the first *M*. *ulcerans* colony was 8 ± 0 days on Middlebrook 7H11, 6.34 ± 0.75 days on *A*. *gracilis*-enriched medium (p<0.01), 6 ± 1 days on *E*. *africanus*- and *V*. *torta*-enriched media (p<0.01), 6 ± 0 days on *V*. *nana*-enriched medium (p<0.01), and 5.67 ± 0.47 days on *C*. *calamistratum*-enriched medium (p<0.01).

### Growth yield

For *M*. *marinum*, *A*. *gracilis*-enriched medium and *C*. *calamistratum*-enriched medium significantly increased the number of detected colonies at each time point compared to Middlebrook 7H11 medium (p<0.05). *E*. *africanus*-enriched medium significantly increased the number of detected colonies starting from day 5 PI (p<0.05) (Fig 1).

**Fig 2:**
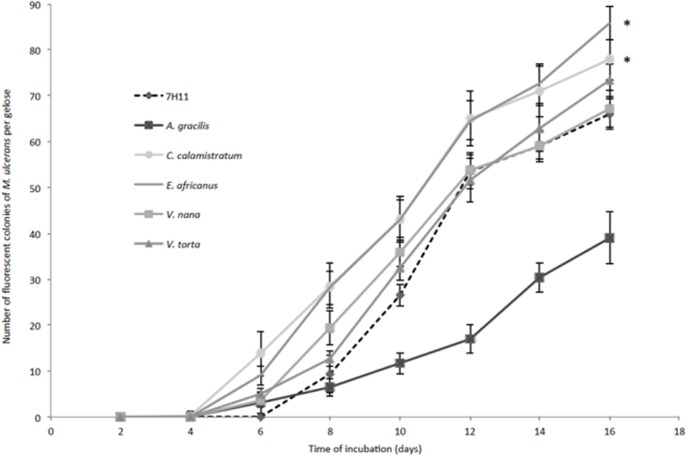
*C*. *calamistratum* and *E*. *africanus* extracts increase the growth rate of *M*. *ulcerans* on Middlebrook 7H11-enriched media. Kinetics of growth of *M*. *ulcerans* in Middlebrook 7H11 medium and Middlebrook 7H11-based solid media enriched with tropical plant extracts (10%, vol:vol), detected by autofluorescence. Each data point represents the mean ± standard error for six plates per time point. The asterisks represent media facilitating a significant increase of the number of *M*. *ulcerans* colonies at each time point compared to Middlebrook 7H11 medium.

Similarly, *C*. *calamistratum*- and *E*. *africanus*-enriched media significantly increased the number of detected *M*. *ulcerans* colonies at each time point compared to Middlebrook 7H11 medium (p<0.05) (Fig 2). *V*. *nana*- and *V*. *torta*-enriched media significantly increased the number of detected colonies starting from day 6 PI and day 10 PI, respectively (p < 0.001). *A*. *gracilis*-enriched medium significantly decreased the number of detected colonies starting from day 8 PI (p<0.05).

## Discussion

In this study, we took advantage of mycobacterial autofluorescence to detect *M*. *marinum* and *M*. *ulcerans* microcolonies. Autofluorescence has been previously described for *Mycobacterium tuberculosis* and *M*. *marinum* [19,20], but this is the first report of autofluorescence for *M*. *ulcerans*. Here, we validated this new method of detection of *M*. *ulcerans* microcolonies, following our recent validation for *Mycobacterium tuberculosis* [20]. Indeed, using autofluorescence significantly speeds the detection of microcolonies. Detecting fluorescence of mycobacterial colonies on plant extract-enriched media was challenging because most plants are autofluorescent [21] due to the presence of chlorophyll [22,23]. Nevertheless, microcolonies of mycobacteria were distinguishable from the fluorescent background because chlorophyll emits autofluorescence at > 600 nm wavelength, whereas mycobacteria emit autofluorescence at 450–550 nm [19]. These data suggest that small colonies of *M*. *ulcerans* and *M*. *marinum* can be detected using fluorescence lighting thanks to the natural autofluorescence of these mycobacteria. The fastidiousness of *M*. *ulcerans* culture makes this procedure of particular interest from a diagnostic perspective.

Using this protocol, we observed that two tropical plant extracts significantly stimulated the growth of *M*. *ulcerans* and *M*. *marinum*. *C*. *calamistratum* and *E*. *africanus* were native to Cameroon before they became endemic in Central Africa, which is the region with the highest number of Buruli ulcer cases in the world [24,25]. Likewise, *V*. *nana* and *V*. *torta* exhibited a significant stimulating effect on the growth of *M*. *ulcerans*. *V*. *nana* is found in Queensland (Australia), and *V*. *torta* is found in Japan and southeast Asia, which are regions where Buruli ulcer cases have been described [26] or suspected. Therefore, some plants that are present in regions where Buruli ulcer is also endemic stimulate the growth of *M*. *ulcerans*. These data agree with the previously reported observation that some aquatic plants stimulate growth and biofilm formation of *M*. *ulcerans* [12].

The characterization of growth-stimulating factors contained in the plant extracts was beyond the scope of the present study. These factors are thermoresistant, as the stimulating extracts have been autoclaved prior to their incorporation into the culture medium. These factors are most likely specific, as we observed that the growth kinetics differed between plants, and *A*. *gracilis* even significantly decreased the growth of *M*. *ulcerans*.

In this study, only one isolate of *M*. *marinum* and one isolate of *M*. *ulcerans* have been tested, and the data reported here should be regarded as preliminary. However, the similar results obtained from both *M*. *marinum* and *M*. *ulcerans* suggest that these data may be extrapolated to *M*. *ulcerans* at large, as *M*. *ulcerans* originated from *M*. *marinum* [3]. These data are aligned with the research priorities identified by WHO [27], *i*.*e*., environmental ecology and transmission mode(s) of *M*. *ulcerans*. These data suggest that *C*. *calamistratum* and *E*. *africanus* extracts could be used as adjuvants to the Middlebrook medium for cultivating *M*. *marinum* and *M*. *ulcerans* from environmental and clinical sources.

## Supporting Information

S1 File Synopsis of characteristics of five aquatic plants used in the study. (DOCX)
