# Supplementary material for: Tropical Plant Extracts Modulating the Growth of Mycobacterium ulcerans
Source: PLoS One. 2015 Apr 23;10(4):e0124626. doi: 10.1371/journal.pone.0124626 (PMC4408112; doi:10.1371/journal.pone.0124626)
Supplement: S1 File — (DOCX) [file pone.0124626.s001.docx]

***Crinum calamistratum***

*Crinum calamistratum* is an aquatic bulbous plant exhibiting dark-green, very narrow but long leaves. Its phylogenetic position is precised in the tree below, based on 5S spacer sequence.


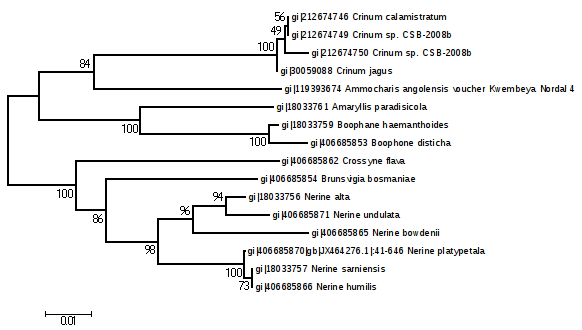


***Vallisneria nana***

*Vallisneria nana* is an aquatic, 15-cm plant exhibiting fine dark-green leaves in rosette. Its phylogenetic position is precised in the tree below, based on 5S spacer sequence.


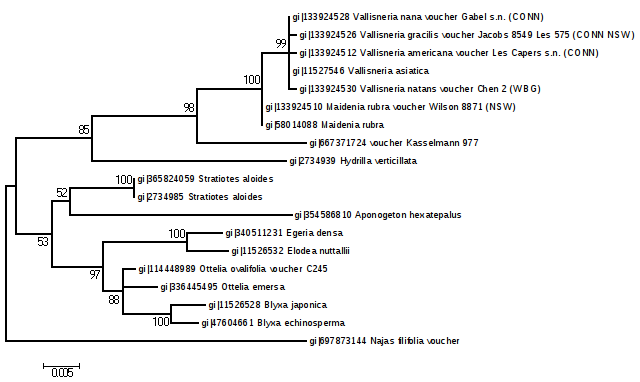


***Echinodorus africanus***

*Echinodorus africanus* is an aquatic plant exhibiting long thin leaves, looking similar to *Echinodorus amazonicus*. No sequence available for phylogenetic relationships exhibition.

***Ammannia gracilis***

*Ammannia gracilis* is an aquatic marsh plant found throughout West Africa. No sequence available for phylogenetic relationships exhibition.

***Vallisneria torta***

*Vallisneria torta* is an aquatic plant exhibiting long thin green leaves. No sequence available for phylogenetic relationships exhibition.
